# Characterization of a Monogamous California Mouse Model of Chemotherapy

**DOI:** 10.1523/ENEURO.0159-25.2025

**Published:** 2025-09-18

**Authors:** Melina M. Seng, Zoe M. Tapp, Erica R. Glasper, Leah M. Pyter

**Affiliations:** ^1^Institute for Behavioral Medicine Research, College of Medicine, The Ohio State University Wexner Medical Center, Columbus, Ohio 43210; ^2^Departments of Neuroscience, College of Medicine, The Ohio State University Wexner Medical Center, Columbus, Ohio 43210; ^3^Psychiatry and Behavioral Health, College of Medicine, The Ohio State University Wexner Medical Center, Columbus, Ohio 43210

**Keywords:** behavior, fatigue, inflammation, neuroinflammation, paclitaxel, *Peromyscus californicus*

## Abstract

Chemotherapy can cause debilitating behavioral side effects (e.g., fatigue, depression, cognitive decline); however, having an intimate partner can buffer these effects. The California mouse (*Peromyscus californicus*) is a rare monogamous mouse species that offers a novel opportunity to model human intimate partnership to identify the neurobiological mechanisms by which mate bonding reduces chemotherapy-associated behavioral side effects. As a first step toward this goal, this pilot study aimed to develop the first chemotherapy model, to our knowledge, in adult male and female California mice. Following a repeated paclitaxel chemotherapy regimen, well characterized in laboratory mice (*Mus musculus*), gross sickness physiology was first assessed after various doses. The 20 mg/kg paclitaxel dose, injected six times every other day, was the highest tolerable, clinically relevant dose and was characterized by moderate body mass loss and increased spleen mass. Thus, further investigation of the effects of this chemotherapy paradigm on peripheral and neural inflammatory gene expression, based on previous reports in laboratory mice, was undertaken; results were mixed. Consistent across the spleen, hippocampus, and hypothalamus, some proinflammatory genes were unexpectedly decreased with chemotherapy (*Il1β*, *Tnf*), whereas one gene was increased (*Icam1*). Chemotherapy also increased fatigue and sociability, but not anxiety-like behavior or cognition. Taken together, this pilot study characterized a translational model of chemotherapy in California mice with clinically relevant gross physiological changes and modest changes in neuroinflammation and behavioral side effects. This work also highlights the need for comparative studies and the growth of research tools for this socially relevant mouse species.

## Significance Statement

The California mouse, a nontraditional laboratory rodent species, displays strict monogamy and offers a unique opportunity to controllably model partnership effects on behavioral health. Utilizing California mice as a model for studying human health and disease is emerging. This study is the first, to our knowledge, to develop a chemotherapy model in this mouse species, characterized by modest changes in inflammation and fatigue, which recapitulates clinical outcomes. This model enables future studies to investigate the biological mechanisms by which partnership reduces chemotherapy-associated behavioral side effects to inform new interventions and ultimately enhance quality of life for chemotherapy patients.

## Introduction

Research using the monogamous California mouse (*Peromyscus californicus*) as a model for understanding human health and disease has been on the rise (Havighorst et al., 2017). California mice offer unique advantages over traditional laboratory rodent models (*Mus musculus*, i.e., laboratory mouse) due to their natural genetic diversity and being among the <10% of mammalian species that form mate bonds (Kleiman, 1977; Havighorst et al., 2017). In fact, California mice display strict genetic monogamy, characterized by lifelong partner preference, mate guarding, and biparental care (Gubernick and Alberts, 1987; Ribble, 1991; Gubernick and Nordby, 1993). These traits increase the attractiveness of this model over the socially monogamous prairie vole (*Microtus ochrogaster*), which is biparental and forms pair bonds but also engages in extra-pair copulations (McGraw and Young, 2010). Studying the unique mate bond of California mice offers a way to controllably model human partnership effects on mental and physical health. Indeed, the benefits of social connection, and specifically that of an intimate partner, on health are well characterized in humans (Holt-Lunstad et al., 2010; Goldman and York Cornwell, 2023). However, to identify underlying biological mechanisms, particularly those that require investigation of the central nervous system, the California mouse model is particularly useful. One such health context that could benefit from the mechanistic understanding of partnership advantages is addressing the common and debilitating side effects of cancer treatment (e.g., chemotherapy).

Chemotherapy is a mainstay treatment in cancer but can result in various behavioral side effects (e.g., fatigue, depression, cognitive decline) that reduce quality of life for patients and their caregivers. In addition, these side effects can ultimately lead to reductions in treatment dosage and therefore increased mortality (Koppelmans et al., 2012; Vichaya et al., 2015). Notably, intimate partnership in humans improves many of these chemotherapy-associated behavioral side effects (Kudel et al., 2008; Shrout et al., 2021; Seng et al., 2025). The leading hypothesized neurobiological mechanism underlying these chemotherapy-associated behavioral side effects is neuroinflammation (Vichaya et al., 2015; Santos and Pyter, 2018). While most chemotherapeutics do not cross the blood–brain barrier (Angeli et al., 2019), systemic inflammatory cytokines released in response to chemotherapy administration can trigger activation of resident innate immune cells in the brain (i.e., microglia; Grant et al., 2023) and neuroinflammation (Weymann et al., 2014). This neuroinflammatory response is linked to behavioral deficits in chemotherapy models using laboratory mice (Vichaya et al., 2015; Sullivan et al., 2022), and pharmacological reductions in inflammation attenuate these side effects (Masocha, 2014; Grant et al., 2023). Additionally, intimate partnership in humans is associated with lower serum inflammatory mediators (Donoho et al., 2013; Jaremka et al., 2020; Shrout et al., 2020), which could be contributing to the buffering of chemotherapy behavioral side effects.

The long-term goal of this project is to use California mice to help identify the biological mechanisms by which mate bonding/partnership reduces chemotherapy-associated behavioral side effects to inform new interventions and ultimately enhance quality of life for chemotherapy patients. As a first step toward this goal, the present pilot study aimed to develop a chemotherapy model in California mice for the first time. It was hypothesized that a repeated paradigm of a common chemotherapeutic, paclitaxel, increases peripheral and central inflammation and sickness behavior in this species, similar to what has been observed in the traditional, non-mate bonding laboratory mouse model (Loman et al., 2019; Grant et al., 2021; Sullivan et al., 2022).

## Materials and Methods

### Animals

Virgin male and female California mice (*P. californicus*), aged 9–20 months [mean, 15.6 months; standard error of the mean (SEM), 0.6] and descendants of mice from the *Peromyscus* Genetic Stock Center (University of South Carolina), were housed in a temperature-controlled (22 ± 3°C) mouse vivarium with a 14:10 light/dark cycle. All mice were given corn cob bedding, cotton nestlets, and standard rodent chow (Harlan 7912) available *ad libitum*. Mice were group-housed, by treatment, 2–3/cage with same-sex cage mates except for the second cohort in Experiment 3, in which some of the mice were singly housed with an additional enrichment item (i.e., a red hut). Mice were mixed sex, except for the 5 and 10 mg/kg chemotherapy groups in Experiment 1, which were all male. Given the sample size, this study was not powered to assess sex differences, except in Experiment 3. All experiments were approved by The Ohio State University Institutional Animal Care and Use Committees and carried out in accordance with the National Institutes of Health Guide for the Care and Use of Laboratory Animals. All efforts were made to minimize animal suffering and to reduce the number of mice used.

### Experimental overview

#### Experiment 1: dose response curve

Mice were used to assess a dose response curve (5, 10, 15, 20, 30 mg/kg) to paclitaxel chemotherapy. Mice were killed 6 h after the last vehicle or chemotherapy injection, and their spleens were weighed. Outcomes from this experiment include body mass (*n* = 28 for vehicle, *n* = 20 for 20 mg/kg, and *n* = 3–4 for the 5, 10, 15, and 30 mg/kg groups) and spleen mass (*n* = 15 for vehicle, *n* = 10 for the 20 mg/kg, and *n* = 3–4 for the 5, 10, and 15 mg/kg groups). Body mass and spleen mass data from Experiments 2 and 3 (when collected 6 h following the final chemotherapy injection) were included in these analyses.

#### Experiment 2: peripheral and central inflammatory gene expression

The same mice that received vehicle or 20 mg/kg chemotherapy, as described in Experiment 1, were used for this Experiment. An open-field test (OFT) was conducted 4–5 h after the final injection. In one subset of mice (*n* = 3–4/group), spleens and brain regions were dissected and used for gene expression analysis. In the second subset of mice (*n* = 4–6/group), hemisected brains were used for central gene expression analysis using an nCounter Inflammation Panel.

#### Experiment 3: chemotherapy-induced behavioral effects

Two cohorts of mice received either vehicle or 20 mg/kg chemotherapy, and OFT was conducted 4–5 h after the final injection. In the first cohort (*n* = 5–6/group), the sucrose splash test (SST) was conducted 1 d after the final injection, and the novel object location and recognition (NOLR) test was conducted the following day. In the second cohort (*n* = 5–6/group), mice underwent a social investigation test 1–3 d after the final chemotherapy injection. In both cohorts, the body mass data over chemotherapy treatment was included in Experiment 1 analyses.

### Chemotherapy treatment

Paclitaxel chemotherapy (LC Laboratories, catalog #P-9600) was dissolved in Cremophor EL:PBS solution (Millipore-Sigma, catalog #238470-1SET) as previously described (Loman et al., 2019) and administered every other day within a 4 h range of time during the light, inactive cycle for a total of six doses, unless otherwise stated. While paclitaxel is used to treat multiple types of cancer (e.g., ovarian, lung, pancreatic; Mekhail and Markman, 2002), this paradigm was modeled after breast cancer patients who receive 4–8 doses of chemotherapy spaced 1–3 weeks apart and was then scaled down to account for the shorter lifespan of mice. A dose response curve was generated using 5, 10, 15, 20, or 30 mg/kg chemotherapy or vehicle injections (100–200 μl, i.p.) based on previous reports in laboratory mice (Loman et al., 2019). In Experiment 1, the mice in the 5 and 10 mg/kg chemotherapy groups received eight injections every other day due to a lack of gross body mass loss. Additionally, due to excessive weight loss, the 30 mg/kg group only received two injections, spaced 5 d apart. The first cohort in Experiment 3 received five injections because most of the chemotherapy mice were approaching 20% loss in body mass after the fifth injection. Mice were pseudorandomized into vehicle or chemotherapy groups based on initial body mass. Body mass was measured every other day, and mice were killed if they lost >20% of their baseline body mass. Two total mice were killed, one from the chemotherapy-treated group in each cohort of Experiment 3. Mice did not have a tumor, so that the effect of chemotherapy independent of a tumor could be studied.

### Reverse transcription-quantitative PCR (RT-qPCR)

In laboratory mice, a similar six-dose paclitaxel chemotherapy paradigm increases inflammatory gene expression [e.g., interleukin-1 beta (*Il1β*) and tumor necrosis factor (*Tnf*)] in brain (hippocampus and hypothalamus) and peripheral tissues (Loman et al., 2019). To assess the extent to which inflammatory genes in the periphery and brain were altered in California mice following 20 mg/kg chemotherapy, species-specific RT-qPCR was conducted. Mice were rapidly decapitated 6 h after their final injection of vehicle or chemotherapy. Peripheral (spleen) and central (hippocampus, hypothalamus) tissues were immediately dissected, spleen weights were obtained, and then all samples were flash-frozen. The spleen was used as a proxy of peripheral inflammation due to limitations of immunoassays for cytokine measurement in this species (data not shown). RNA was extracted from spleen and brain tissues using RNeasy Mini Kits (Qiagen, catalog #74106), and then cDNA was reverse transcribed using qScript cDNA SuperMix (Quantabio) as previously described (Otto-Dobos et al., 2024). Gene expression was measured in duplicate using Sybr Green Master Mix (Thermo Fisher Scientific, catalog #A25776) with custom-designed primers and amplified using a QuantStudio 5-Real Time PCR System (Thermo Fisher Scientific). Cycling parameters were optimized for the custom-designed primers as follows: UDG activation (50°C, 2 min), initial denaturation (95°C, 2 min), and then 40 cycles of (1) denaturation (95°C, 15 s), (2) anneal (55°C, 15 s), and (3) extend (72°C, 1 min), followed by the default dissociation steps.

Primers specific to the California mouse genome [*Il1β*, *Tnf*, intercellular adhesion molecule 1 (*Icam1*), actin beta (*Actb*), and beta-2-microglobulin (*b2m*)] were generated by BLASTing the gene of interest against *P. californicus* mRNA sequences using the National Center for Biotechnology Information website (Pyter et al., 2005; Hyer et al., 2017). Primer sequences were separated by at least one intron, when possible, on the corresponding genomic DNA, and primers were created by Integrated DNA Technologies with standard desalting and no formulation. A four- to five-point standard curve was run on every plate, and primer efficiencies were determined to be between 90 and 110%. Gene expression was determined relative to the curve and then the geometric mean of the endogenous control genes (*b2m* and *Actb*). Fold change was then determined by dividing by the average of the vehicle groups. The primer sequences are listed in Extended Data Figure 2-1.

#### nCounter inflammation panel

To screen for additional neuroinflammatory transcriptional changes, we ran a 200-gene laboratory mouse inflammatory panel. Following rapid decapitation 6 h after the final vehicle or 20 mg/kg chemotherapy injection, brain tissue with the cerebellum removed was hemisected and flash-frozen on dry ice. RNA was extracted as described above and then ran on the (laboratory) Mouse Inflammation nCounter panel (NanoString Technologies, catalog #115000082) via the OSU Comprehensive Cancer Center (OSU CCC) Genomics Shared Resource Facility. Genes with mean raw mRNA counts <20 were removed, and the rest were normalized to three endogenous controls (*Cltc*, *Gapdh*, and *Pgk1*) using the nCounter Analysis Software (NanoString Technologies). Analyses were conducted using the DESeq2 R package (version 4.4.1). Heatmaps were made with the R package pheatmap (v1.0.12) with each box representing the standardized *Z*-score of normalized mRNA counts within vehicle or chemotherapy groups.

Given that this panel was designed for laboratory mice, all genes identified as having significant differential expression between treatment groups, based on the unadjusted *p* value, were next validated using RT-qPCR with California mouse-specific custom primers as described above. Efficiencies of quantifiable genes were between 90 and 110%, except for *Hdac4* which had an efficiency of 118%. The primer sequences are listed in Extended Data Figure 2-1.

### Behavioral testing

In laboratory mice, paclitaxel chemotherapy induces behavioral deficits (Loman et al., 2019; Sullivan et al., 2022). Thus, the following tests were selected to investigate chemotherapy-induced fatigue, depressive-like behaviors, and cognitive and sociability deficits following vehicle or 20 mg/kg chemotherapy injections in California mice. All behavioral tests occurred during the dark, active phase and mice were acclimated to the behavioral space >30 min before testing. To minimize stress from separation, cage mates were tested concurrently in adjacent apparatuses. White noise was used to obscure background noise. Behavioral apparatuses were cleaned with 70% ethanol between mice.

#### OFT

A 10 min OFT was conducted as previously described (Loman et al., 2019). Briefly, mice were placed in one corner of a 16 × 16 in. plexiglass arena and were allowed to explore for 10 min. Measurements of fatigue (total distance traveled, resting time) and anxiety-like behavior (central tendency) were recorded and analyzed using an automated system of 16 photobeams across two dimensions (PAS Data Reporter, San Diego Instruments).

#### SST

In the SST, mice were sprayed twice with a 10% sucrose solution on their dorsal coat and then placed in a new, empty cage under red light, and their behavior was observed for 5 min. Grooming duration was recorded to assess self-care, with reduced grooming time interpreted as depressive-like behavior (Frisbee et al., 2015).

#### NOLR test

An NOLR test was conducted, similar to previously described (Strehle et al., 2024). Mice were habituated to the arena twice prior to the test, first via the OFT and again 1 d later following the completion of the SST. The NOLR test consisted of a 5 min familiarization trial, followed by a 15 min intertrial interval when the mice were placed back in their home cage and then a 5 min memory trial. Two familiar objects (50 ml centrifuge tubes filled with sand) were placed in adjacent corners. Objects were cleaned with 70% ethanol, and one familiar object was placed in the same location, while a novel object (marble) was placed in a novel corner. The discrimination index was calculated: (time investigating novel object − time investigating familiar object) / total time investigating.

#### Social investigation test

A three-chamber social investigation test, adapted from Kaidanovich-Beilin et al. (2011) and Walker et al. (2023), was conducted. On all days, mice were placed in the center chamber of an acrylic, three-chambered arena (15.5 × 23.5 × 9 in.; Noldus Information Technology,Leesburg, Virginia) with doors to the other arenas closed and allowed to explore for 2 min. Then the doors separating the center chamber from the outer chambers were removed, and the mice were able to explore all three chambers for 10 min on Days 1–2 and 15 min on Day 3. On Day 1, an acrylic holding cage (diameter, 3.9 in.; height, 7.9 in.) was placed in each of the outer chambers. On Day 2, a 50 ml centrifuge tube filled with paper was placed in the acrylic holding cages in each of the outer chambers. Lastly, on Day 3, a novel object (large LEGO piece; length, 5 in.; height, 2.2 in.) was placed in an acrylic holding cage in one of the outer chambers and a novel, same-sex conspecific mouse was placed in an acrylic holding cage in the other outer chamber. The percentage of time spent investigating all chambers was recorded, with more time spent investigating the novel mouse compared with the nonsocial stimulus (novel object) indicating greater sociability.

### Statistical analysis

Statistical analyses were conducted using GraphPad Prism software (versions 8.3.0 and 10.0.3, GraphPad Software). Statistical analysis of body mass was performed using a mixed-effect model and controlling for multiple comparisons using Tukey's test. Statistical analysis of spleen mass was performed using a one-way analysis of variance and controlling for multiple comparisons using Tukey's test. Statistical analyses of all gene expression and behavioral tests were conducted using a parametric, unpaired *t* test, except for Figure 3*D* which was conducted using a two-way ANOVA and controlling for multiple comparisons using Tukey's test. According to the Grubbs test, one outlier from the vehicle group was removed from the hippocampus *Il1β*, hypothalamus *Icam1*, and spleen *Il1β* gene expression analyses, one sample from the chemotherapy group measuring hippocampal *Il1β* was excluded due to high cycle threshold difference, and one outlier from the vehicle group was removed from the *Map2k1* RT-qPCR validation. Analyses assessing familial relatedness and sex effects in Experiment 3 were conducted using linear mixed-effect and linear models, respectively, in R (version 4.5.1). Familial relatedness was defined by grouping mice from the same parental pair and was incorporated as a random effect in the model; familial relatedness was unable to be determined in three mice. Data were determined to be statistically significant at *p* < 0.05, and all graphs are presented as mean ± SEM using GraphPad.

## Results

### California mice respond to paclitaxel chemotherapy in a dose-dependent manner

**Figure 1.**
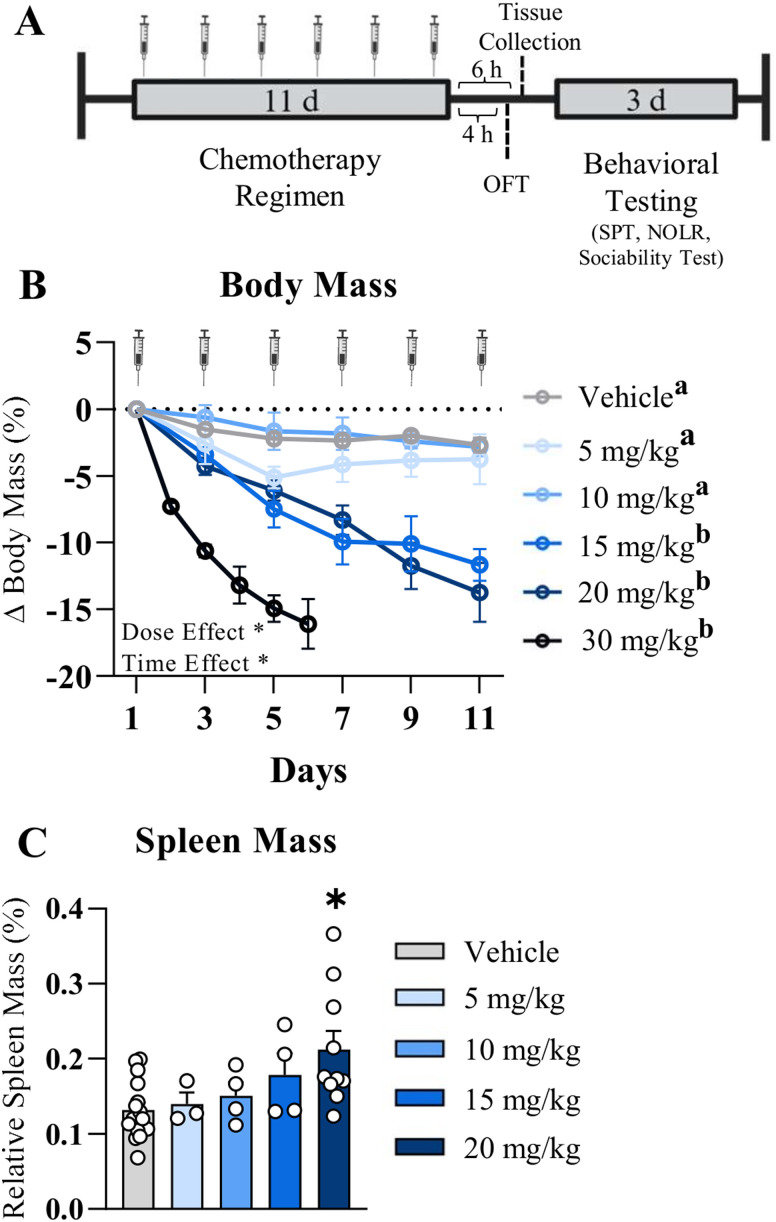
Study design and physiology. ***A***, Experimental overview, (***B***) percentage change in body mass over time from baseline, and (***C***) spleen mass as a percentage of total body mass. Results are shown as mean ± SEM. For ***B***, *n* = 28 for vehicle, *n* = 20 for 20 mg/kg chemotherapy, and *n* = 3–4 for the 5, 10, 15, and 30 mg/kg chemotherapy groups. * is the main effect with *p* < 0.05. Groups with nonoverlapping subscript letters “a,b” are statistically different from each other over time (*p* < 0.05). For ***C***, *n* = 15 for vehicle, *n* = 10 for the 20 mg/kg chemotherapy, and *n* = 3–4 for the 5, 10, and 15 mg/kg chemotherapy groups. *p < 0.05 compared with vehicle. Syringe symbols in ***A*** and ***B*** represent chemotherapy injections.

Adult California mice were repeatedly injected with one of several doses of vehicle or paclitaxel chemotherapy (5, 10, 15, 20, 30 mg/kg; Fig. 1*A*). Using mice from Experiments 1–3, percentage body mass change from baseline was assessed to determine a dose–response curve for potential chemotherapy-induced weight loss (Loman et al., 2019) in this unique species. Paclitaxel reduced body mass overall (Fig. 1*B*; main effect of treatment, *F*_(5,58)_ = 15.9; *p* < 0.001), driven primarily by the loss in the 15, 20, and 30 mg/kg groups compared with vehicle (*p* < 0.01 for all). Indeed, the 30 mg/kg group lost a substantial amount of body mass after two out of six injections and was unable to continue to receive the full chemotherapy regimen. Nevertheless, the 30 mg/kg group lost more weight over time than the vehicle, 5, and 10 mg/kg groups (*p* < 0.0001, *p* < 0.01, and *p* < 0.001, respectively). The 15 and 20 mg/kg groups lost more weight over time compared with the vehicle and 10 mg/kg groups (*p* < 0.01 and *p* < 0.05; and *p* < 0.0001 and *p* < 0.01, respectively). There also was a significant main effect of time on paclitaxel-induced weight loss (main effect of time, *F*_(5, 353)_ = 36.24; *p* < 0.0001). Given that paclitaxel causes splenomegaly in laboratory mice (Loman et al., 2019), final spleen mass was compared as a gross assessment of systemic immune activation. In California mice, paclitaxel also increased normalized spleen mass, particularly in the 20 mg/kg group (Fig. 1*C*; *p* < 0.01) compared with that in the vehicle group. Spleen mass of the 30 mg/kg group was not included due to only receiving two chemotherapy injections. Due to the 20 mg/kg chemotherapy dose being the highest tolerable dose, without extreme weight loss and recapitulating splenomegaly, subsequent experiments included only the 20 mg/kg chemotherapy and vehicle groups.

### Chemotherapy treatment modestly alters peripheral and central inflammation

**Figure 2.**
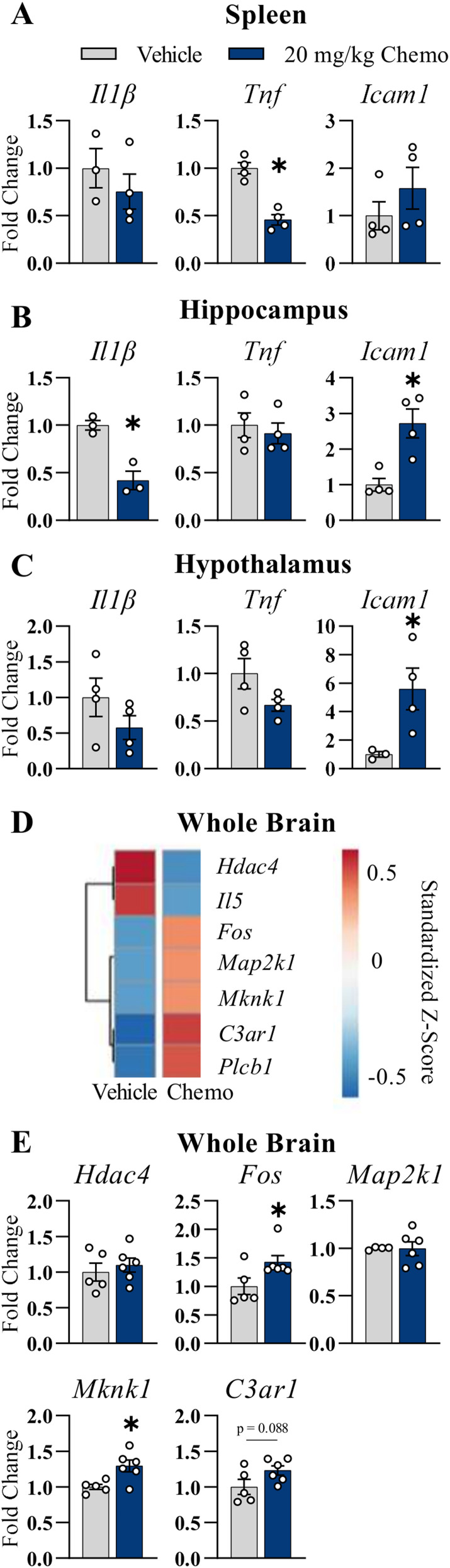
Peripheral and central inflammatory gene expression. Fold change in (***A***) splenic (***B***) hippocampal and (***C***) hypothalamic *Il1β*, *Tnf*, and *Icam1* gene expression 6 h following the final vehicle or 20 mg/kg chemotherapy injection relative to vehicle controls. ***D***, Heatmap of standardized *Z*-score with genes significantly altered in the hemisected cerebral brain tissue by 20 mg/kg chemotherapy compared with vehicle 6 h following the final injection (red, increased; blue, decreased). ***E***, Fold change of inflammatory gene expression in the cerebral brain tissue validated by species-specific RT-qPCR. Results are shown as mean ± SEM. For ***A–C***, *n* = 3–4/group; for ***D***, ***E***, *n* = 4–6/group. *p < 0.05 compared with vehicle. The *P. californicus* primer sequences are listed in Extended Data Figure 2-1.

To assess the potential acute inflammatory effects of chemotherapy treatment in California mice, we preliminarily assessed inflammatory gene expression in the periphery (spleen) and brain regions that regulate sickness, affective-like, and cognitive behavior (hippocampus, hypothalamus) 6 h after the last injection. The spleen was used as a proxy of peripheral inflammation due to limitations in detectability for plasma using laboratory mouse immunoassays (data not shown). In contrast to our hypothesis based on previous studies of paclitaxel in laboratory mice, the 20 mg/kg chemotherapy paradigm decreased mRNA expression of proinflammatory cytokines, *Tnf*, in the spleen (Fig. 2*A*; *p* < 0.001) and *Il1β* in the hippocampus (Fig. 2*B*; *p* < 0.01) compared with that in the vehicle group. Conversely, the 20 mg/kg chemotherapy treatment increased gene expression of *Icam1* in the hippocampus and hypothalamus (Fig. 2*B*,*C*; *p* < 0.01 and *p* < 0.05, respectively). Though not all statistically significant, it was noted that patterns consistent with these findings were observed in virtually each tissue (Fig. 2*A*–*C*; *p* > 0.05).

10.1523/ENEURO.0159-25.2025.f2-1 Figure 2-1 *P. Californicus* Primer Sequences. Download Figure 2-1, DOCX file.

To screen for additional inflammatory marker differences in the brain, we performed a comprehensive inflammatory laboratory mouse gene expression panel using hemisected brains from the vehicle and 20 mg/kg groups. Heatmaps show the mean standardized *Z*-score between groups, and 7 genes out of the 200-gene panel were significantly different (Fig. 2*D*; *p* < 0.05). *Hdac4* and *Il5* gene expression was significantly decreased by chemotherapy compared with vehicle, whereas *fos*, *Map2k1*, *Mknk1*, *C3ar1*, and *Plcb1* were increased (Fig. 2*D*; *p* < 0.05 for all). To validate the genes identified by this screen in a species-specific manner, we performed RT-qPCR on the same RNA used for the panel. Increased gene expression of *Fos*, *Mknk1*, and a trend in *C3ar1* gene expression was replicated using species-specific RT-qPCR (Fig. 2*E*; *p* < 0.05, *p* < 0.05, and *p* = 0.09, respectively). However, there were no significant differences between the vehicle and chemotherapy groups in *Hdac4* and *Map2k1* expression (Fig. 2*E*; *p* > 0.05 for both). Due to low expression in the brain, *Il5* and *Plcb1* gene expression were not quantifiable via RT-qPCR.

### Chemotherapy treatment induces fatigue and sociability but not anxiety-like behavior or cognitive deficits

**Figure 3.**
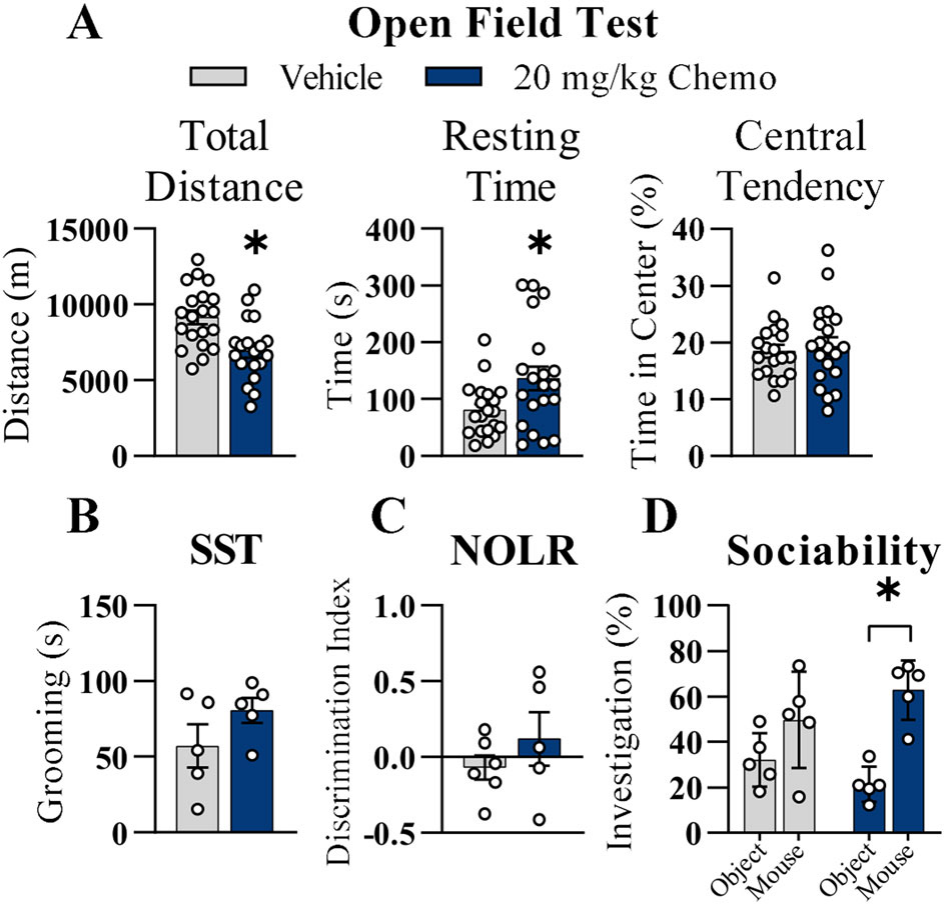
Chemotherapy-induced behavioral side effects. ***A***, Total distance traveled, resting time, and percentage central tendency in a 10 min OFT 4–5 h following the final vehicle or 20 mg/kg chemotherapy injection. ***B***, Grooming duration during the 5 min SST, (***C***) discrimination index during the 5 min memory trial of the NOLR test, and (***D***) the percentage of time investigating a novel object or a novel mouse during the 15 min social investigation test. For ***B–D***, behavioral testing occurred 1–3 d following the final chemotherapy or vehicle injections in two cohorts. For ***A***, *n* = 20/group; for ***B–D***, *n* = 5–6/group. Results are shown as mean ± SEM. *p < 0.05 compared with vehicle.

To assess potential behavioral deficits following chemotherapy treatment, a battery of behavioral tests was conducted over multiple cohorts of mice 0–3 d following the last injection. In the OFT, California mice that received 20 mg/kg injections of paclitaxel traveled a shorter distance and rested longer compared with the vehicle group (Fig. 3*A*; *p* < 0.01 and *p* < 0.05, respectively). There were no differences in central tendency, a measure interpreted as anxiety-like behavior (*p* > 0.05). When familial relatedness, as defined by grouping mice from the same parental pair, was included in the model, the original relationships held, indicating that hereditary variation is not driving the observed chemotherapy-induced behavioral deficits. Additionally, while sex had a significant effect on resting time and central tendency, such that males rested less and spent less time in the center, it did not change the significance of the treatment effects in the original models.

Furthermore, there were no significant differences in grooming duration in the SST (Fig. 3*B*; *p* > 0.05) or in the discrimination index during the memory trial of the NOLR test (Fig. 3*C*; *p* > 0.05). In the social investigation test, the 20 mg/kg chemotherapy group investigated the novel mouse significantly more than the novel object (Fig. 3*D*; *p* < 0.01), indicating greater sociability. There were no statistically significant differences in the percentage of time the vehicle group investigated the novel object versus the novel mouse (Fig. 3*D*; *p* > 0.05).

## Discussion

The present pilot study developed a chemotherapy model, for the first time, in a genetically monogamous species, the California mouse (*P. californicus*). Consistent with our hypothesis, a repeated paradigm of a common chemotherapeutic, paclitaxel, caused modest sickness behavior (weight loss, fatigue), similar to that observed in the traditional mouse model, *M. musculus* (Ray et al., 2011; Loman et al., 2019; Sullivan et al., 2022). In contrast, mixed neuroinflammatory results were observed in concert with the absence of affective-like or cognitive deficits in the tests performed. Importantly, this study sets into motion the potential for using California mice to study the mechanisms by which mate bonding attenuates chemotherapy-induced sickness behaviors.

Here, a dose–response curve using a clinically relevant, repeated dosing paradigm resulted in moderate sickness physiology (∼14% weight loss) and behavioral (∼24% reduction in locomotion) outcomes, comparable with common clinical outcomes (Berger et al., 2015), only with the 20 mg/kg dose. While this dose was the highest tolerated in this species, paclitaxel dosing in rodent models can vary, often depending on the outcome of focus. The most comparable laboratory mouse paradigm, only differing in the dose, consists of 30 mg/kg paclitaxel every other day for six doses and results in modest sickness behavior (e.g., ∼6% weight loss and ∼19% reduction in OFT locomotion; Loman et al., 2019; Sullivan et al., 2022). Other studies using 8–20 mg/kg paclitaxel, administered every other day for 4–12 doses, have also reported chemotherapy-induced behavioral deficits (e.g., fatigue, cognition, affective-like behavior; Huehnchen et al., 2017; Toma et al., 2017; Chang et al., 2020), though less robust than the 30 mg/kg paclitaxel dose. Conversely, models using *Rattus norvegicus* or focusing on nociceptive behavior in laboratory mice often use a lower dose, 2 mg/kg paclitaxel, administered for 4–5 doses (Kalynovska et al., 2020; Martínez-Martel et al., 2022). Altogether, the dose and frequency can vary depending on the model, though repeated paradigms are more comparable with clinical regimens than a single injection (Perez, 1998). Furthermore, the increased sensitivity of stress of the California mice (Glasper and Devries, 2005; Harris et al., 2012), particularly in response to repeated handling and injections, could explain their intolerance to higher doses such as 30 mg/kg paclitaxel.

The inflammatory gene expression results from the spleen and brain regions that regulate behavioral side effects (hippocampus and hypothalamus) measured acutely after 20 mg/kg chemotherapy were mixed, with decreases in some proinflammatory genes (*Il1β*, *Tnf*) and an increase in one gene involved in leukocyte trafficking (*Icam1*). These results largely contradict similar studies using laboratory mice which report transiently increased proinflammatory *Il1β* and *Tnf* gene expression in various brain regions and in the blood 6 h after chemotherapy (Loman et al., 2019; Grant et al., 2021; Sullivan et al., 2022); however, one laboratory mouse study reports a decrease in neuroinflammation and no change in plasma cytokines 24 h after chemotherapy (Chang et al., 2020). Differences within these laboratory mouse studies could be attributed to differences in administration timing, dosage, sex, and outcomes. Decreased gene expression of *Il1β* in the hypothalamus observed 11 d after chemotherapy (Sullivan et al., 2022) was hypothesized to be due to delayed local immune cell inactivation or death (Loman et al., 2019). These differences in inflammatory responses between laboratory mice and California mice may also be attributed to potential differences in immune responses, as paclitaxel is a toll-like receptor 4 agonist (Byrd-Leifer et al., 2001). Indeed, *Peromyscus* species have varied immune responses based on their social strategies (Martin et al., 2006) and also have much higher concentrations of glucocorticoids than laboratory mice (Harris et al., 2012), which are classically anti-inflammatory (Coutinho and Chapman, 2011).

Next, a comprehensive screening of additional inflammatory genes in the hemisected cerebral tissue corroborated the limited effects of paclitaxel on the brain markers assessed. A few genes increased in gene expression (*fos*, *Map2k1*, *Mknk1*, *C3ar1*, *Plcb1*), and two others decreased in expression (*Hdac4*, *Il5*) in the 20 mg/kg chemotherapy group compared with vehicle controls. However, this discussion will be limited to the two gene changes (*Fos* and *Mknk1*) that were validated by follow-up species-specific RT-qPCR analyses. *Fos* is a marker of neuronal activation in the brain, and increased expression in rodent brains treated with other chemotherapeutics (e.g., cisplatin) reflects activation of neural systems that contribute to chemotherapy-induced side effects (e.g., nausea, vomiting, weight loss; Horn et al., 2007). In the present study, it is possible that *Fos* was activated in response to the observed weight loss; however, further work is needed to identify the specific brain regions and underlying causes. Additionally, *Mknk1* is involved in regulating inflammatory responses (Joshi and Platanias, 2012) and is differentially expressed in the periphery (e.g., blood, dorsal root ganglia) in breast cancer survivors and mice with paclitaxel-induced peripheral neuropathy compared with controls (Kober et al., 2020). The whole cerebral tissue was used for this inflammatory panel instead of specific dissected brain regions, which may explain the absence of gene expression differences in *Il1β*, *Tnf*, and *Icam1* observed in the earlier experiment. Overall, this transcriptional work highlights the need for the expansion of transcriptomic/proteomic resources for California mice and other valuable nontraditional models (Havighorst et al., 2017). It also may reflect the notable paucity of information about the immune system in this species (Havighorst et al., 2017). Indeed, multiple mouse cytokine immunoassays using plasma from this species have been remarkably undetectable (data not shown).

Lastly, behavioral side effects that are commonly reported in both chemotherapy patients and laboratory mouse models (Santos and Pyter, 2018; Loman et al., 2019; Sullivan et al., 2022) were assessed in this study. Consistent with our hypothesis, chemotherapy increased fatigue, independent of sex and familial relatedness, recapitulating the most common behavioral side effect in cancer patients (Berger et al., 2015) and consistent with similar laboratory mice studies (Ray et al., 2011; Sullivan et al., 2022). In contrast, anxiety-like behavior was not observed, though it is also not consistently recapitulated in laboratory mice studies (Huehnchen et al., 2017; Loman et al., 2019). No cognitive differences were observed between vehicle and chemotherapy groups, which contradicts findings in laboratory mice (Huehnchen et al., 2017; Chang et al., 2020) but may be due to differences in the behavior tests used or the time of testing relative to chemotherapy. Chemotherapy slightly increased sociability; however, a preference for the novel mouse was expected in the vehicle group, suggesting that the sample size was insufficient. Other limitations of this project that may influence the interpretations include the variability of sexes, ages, and social housing, in some cases. In contrast, the battery of behavioral tests that were performed is considered a strength.

Altogether, this pilot study is the first to develop a model of chemotherapy in a genetically monogamous species, the California mouse. This new model was characterized by sickness behavior (e.g., weight loss, fatigue) and modest changes in central and peripheral inflammatory markers. This model may be useful for future studies systematically investigating the biological mechanisms by which mate bonding reduces chemotherapy-associated behavioral side effects to inform new interventions and ultimately enhance quality of life for chemotherapy patients.

## Data Availability

Data will be made available upon request.

## Synthesis

Reviewing Editor: Charlotte Cornil, Universite de Liege Decisions are customarily a result of the Reviewing Editor and the peer reviewers coming together and discussing their recommendations until a consensus is reached. When revisions are invited, a fact-based synthesis statement explaining their decision and outlining what is needed to prepare a revision will be listed below. The following reviewer(s) agreed to reveal their identity: Hippokratis Kiaris.

This manuscript has now been reviewed by two reviewers who agrees on the novelty and importance of this pilot study. Establishing a chemotherapy model in California mice establishes the bases for future studies to investigate how all aspects of socially, in particular pair bonding, interact with chemotherapy. The reviewers raised a few aspects that require clarification/ improvement. Importantly, reviewer 2 raises the question of potential sex differences, which should be addressed.

Reviewer 1:

This is an interesting manuscript investigating how chemotherapy impacts physiology and behavior in a monogamous rodent. Previous work in humans and animals show that while chemotherapy can play a life-saving role in treating cancer, it can have debilitating effects on behavior. In humans social interactions can help buffer these effects but the mechanisms are unknown. Establishing a chemotherapy model in California mice will provide an opportunity to discover these mechanisms. The experiments are straightforward and well designed.

Specific comments:

Please confirm if the p-values on the panel are adjusted for the false discovery rate.

In experiment 1, why does the 20 mg/kg group have so many more mice than the other groups? We mice from experiments 2 and 3 combined for these analyses?

It would be good for the authors to provide some more details on the Nanostring approach. The text says is a "Mouse" panel. Does that mean the panel is using Mus sequences for Peromyscus? Cytokine sequences tend to be more variable than other genes, did the authors assess the extent to which sequence divergence may have impacted the results? Sequence variation may explain why Mus cytokine immunoassays could not detect signal in Peromyscus samples.

When the authors say "cage mates were tested at the same time" do they mean sequentially?

In figure 1B the labeling made it hard to visualize the dose versus time effects (both had a *).

It seems to me that if not all laboratory mouse studies report chemo-induced inflammation that the current results may not be all that contradictory. Are there similarities between the Chang 2020 paper and the current study compared to the Grant, Loman and Sullivan studies?

Reviewer 2

This is a very important study, the first of its kind, on the effects of chemotherapy in California mice. Besides the interesting data in comparison to the traditional model Mus, it establishes the baseline for future studies that will explore the consequences of social interactions, pair bonding in particular, in cancer treatment and inflammation. Furthermore, it demonstrates that stereotypical responses obtained from experiments in inbred mice may not be relevant to other species, including humans, as evidence by the diversity in the responses in this outbred model. The paper is nicely written and all experiments are appropriately conducted and interpreted. Furthermore, the limitations of this study are outlined in the discussion.

Some points to consider are the following:

1. Likely pedigrees are available for these animals. Taken into account the relatedness of the animals may enable pairwise comparisons of the results and reveal trends that currently are masked.

2. What was the breeding history of these mice? Were they all virgins, breeders? If mixed, does this account for differences?

3. For the experiments that both males and females were used, when numbers allow it, was there a sex difference in the responses?

## Author Response

Response to Reviewer comments:

The authors would like to thank both Reviewers and the Editor for your time and thoughtful review of our manuscript. We have taken care to consider and incorporate the suggested edits to the manuscript. The authors believe that the manuscript has been significantly improved by the comments. The comments are below in black; our responses are in blue.

Reviewer 1:

1. Please confirm if the p-values on the panel are adjusted for the false discovery rate.

We thank the Reviewer for helping to clarify this point. The p-values for the Nanostring panel were not adjusted for the false discovery rate due to the panel not being a species match.

Lines 165-167: Given that this panel was designed for laboratory mice, all genes identified as having significant differential expression between treatment groups, based on the unadjusted p-value, were next validated using RT-qPCR with California mouse-specific custom primers as described above.

2. In experiment 1, why does the 20 mg/kg group have so many more mice than the other groups? Were mice from experiments 2 and 3 combined for these analyses? Yes, the body and spleen mass from Experiments 2 and 3 were combined in the analysis for Experiment 1. We have clarified this point in the Methods.

Lines 92-93: Body mass and spleen mass data from Experiments 2 and 3 (when collected 6 h following the final chemotherapy injection) were included in these analyses.

3. It would be good for the authors to provide some more details on the Nanostring approach. The text says is a "Mouse" panel. Does that mean the panel is using Mus sequences for Peromyscus? Cytokine sequences tend to be more variable than other genes, did the authors assess the extent to which sequence divergence may have impacted the results? Sequence variation may explain why Mus cytokine immunoassays could not detect signal in Peromyscus samples.

We thank the Reviewer for this suggestion. We have added in the Methods that this was a laboratory mouse (aka Mus musculus) panel. Additionally, due to validation of the significantly differentiated genes using species-specific (California mouse) primers, we did not assess the extent to which sequence divergence may have impacted the results.

Lines 157-159: RNA was extracted as described above and then ran on the (laboratory) Mouse Inflammation nCounter panel (NanoString Technologies, Seattle, WA, USA, cat # 115000082) via the [Author University] Genomics Shared Resource Facility.

4. When the authors say "cage mates were tested at the same time" do they mean sequentially? We thank the Reviewer for helping to clarify this point. For every behavioral test, there were multiple behavioral apparatuses where cage mates could be tested concurrently, instead of being tested sequentially.

Lines 175-176: To minimize stress from separation, cage mates were tested concurrently in adjacent apparatuses.

5. In figure 1B the labeling made it hard to visualize the dose versus time effects (both had a *).

We have indicated in the Figure Legends that the asterisk after the dose and time effects are representing main effects, whereas the subscript letters following the treatment groups (e.g., Vehicle, 5 mg/kg, etc...) are indicating significant differences in their interactions. Since both dose and time have a significant main effect, they both have an asterisk in the figure.

Lines 527-528: * = main effect with p<0.05. Groups with non-overlapping subscript letters "a,b" are statistically different from each other over time (p<0.05).

6. It seems to me that if not all laboratory mouse studies report chemo-induced inflammation that the current results may not be all that contradictory. Are there similarities between the Chang 2020 paper and the current study compared to the Grant, Loman and Sullivan studies? We thank the Reviewer for this comment. We have added in additional discussion of this point in the Discussion.

Lines 322-328: These results largely contradict similar studies using laboratory mice which report transiently increased pro-inflammatory Il1β and Tnf gene expression in various brain regions and in the blood 6 h after chemotherapy (Grant et al., 2021; Loman et al., 2019; Sullivan et al., 2022); however, one laboratory mouse study reports a decrease in neuroinflammation and no change in the plasma cytokines 24 h after chemotherapy (Chang et al., 2020). Differences within these laboratory mouse studies could be attributed to differences in administration timing and dosage, sex, and outcomes.

Reviewer 2:

1. Likely pedigrees are available for these animals. Taken into account the relatedness of the animals may enable pairwise comparisons of the results and reveal trends that currently are masked.

We thank the reviewer for this suggestion. We have now accounted for familial relatedness by grouping mice together from the same parental pair. Given the large number of parental pairs, we are only powered to assess relatedness in the OFT (Experiment 3). We have included these findings in Methods, Results, and Discussion.

Lines 224-227: Analyses assessing familial relatedness and sex effects in Experiment 3 were conducted using linear mixed-effects and linear models, respectively, in R (version 4.5.1, Vienna, Austria). Familial relatedness was defined by grouping mice from the same parental pair and was incorporated as a random effect in the model; familial relatedness was unable to be determined in three mice.

Lines 280-285: When familial relatedness, as defined by grouping mice from the same parental pair, was included in the model, the original relationships held, indicating that heredity variation is not driving the observed chemotherapy-induced behavioral deficits.

Lines 358-360: Consistent with our hypothesis, here chemotherapy increased fatigue, independent of sex and familial relatedness, recapitulating the most common behavioral side effect in cancer patients (Berger et al., 2015) and consistent with similar laboratory mice studies (Ray et al., 2011; Sullivan et al., 2022).

2. What was the breeding history of these mice? Were they all virgins, breeders? If mixed, does this account for differences? All mice used in the experiments were virgins, and we have included this in the Methods.

Lines 74-77: Virgin male and female California mice (P. californicus), aged 9-20 months (mean: 15.6 months; SEM: 0.6) and descendants of mice from the Peromyscus Genetic Stock Center (University of South Carolina, Columbia, SC), were housed in a temperature-controlled (22 {plus minus} 3 {degree sign}C) mouse vivarium with a 14:10 light:dark cycle.

3. For the experiments that both males and females were used, when numbers allow it, was there a sex difference in the responses? We are only powered to assess sex differences in the OFT results (Experiment 3). These findings are now included in the Methods, Results, and Discussion.

Lines 81-82: Given the sample size, this study was not powered to assess sex differences, except in Experiment 3.

Lines 224-225: Analyses assessing familial relatedness and sex effects in Experiment 3 were conducted using linear mixed-effects and linear models, respectively, in R (version 4.5.1, Vienna, Austria).

Lines 283-285: Additionally, while sex had a significant effect on resting time and central tendency, such that males rested less and spent less time in the center, it did not change the significance of the treatment effects in the original models.

Lines 358-360: Consistent with our hypothesis, here chemotherapy increased fatigue, independent of sex and familial relatedness, recapitulating the most common behavioral side effect in cancer patients (Berger et al., 2015) and consistent with similar laboratory mice studies (Ray et al., 2011; Sullivan et al., 2022).
