# Supplementary material for: Characterization of a Monogamous California Mouse Model of Chemotherapy
Source: eNeuro. 2025 Sep 18;12(9):ENEURO.0159-25.2025. doi: 10.1523/ENEURO.0159-25.2025 (PMC12453628; doi:10.1523/ENEURO.0159-25.2025)
Supplement: Figure 2-1 — P. Californicus Primer Sequences. Download Figure 2-1, DOCX file. [file eneuro-12-ENEURO.0159-25.2025-s001.docx]

| **Figure 2-1. *P. Californicus* Primer Sequences** | | |  |  |
| --- | --- | --- | --- | --- |
| **Transcript** | **Accession #** | **Forward Primer** | **Reverse Primer** |  |
| *Il1β* | XM_052725849.1 | ATGACAGCTTTCCACAGTGATG | CATCTGGACAGCTCAGGTCA |  |
| *Tnf* | XM_052743832.1 | ACCCTCACACTGAGGTCGT | CTCCAGCTGCTCATCCACTT |  |
| *Icam1* | XM_052753706.1 | GCCTCTGCCACCATCATCTT | AAGAACACCACGGAGAGCTG |  |
| *Actb* | XM_052754162.1 | ATATCGCTGCGCTCGTTGTC | CACCATCACACCCTGATGCC |  |
| *B2m* | XM_006995122.1 | TCTAGTGGGAGGTCCTGTGG | TGCGTTAGACCAGCAGAAGG |  |
| *Il5* | XM_052757813.1 | AGGCTTCCTGTTCCTACCCA | CACACTGCTCTTTTTGGCGG |  |
| *Fos* | XM_052738230.1 | TGGTGAAGACCATGTCAGGC | AGTTGGTCTGTCTCCGCTTG |  |
| *Map2k1* | XM_052712024.1 | AAACCTGCAATCCGGAACCA | CAAGGACCCACCATCCATGT |  |
| *Plcb1* | XM_052724081.1 | CAAGCCAAGATGGCCGAGTA | TACCCCAGATTCCAGTGGGT |  |
| *Mknk1* | XM_052715710.1 | ACGAGTTTCCCGACAAGGAC | TGCTGCTGTTTCTCTGGAGG |  |
| *C3ar1* | XM_052720035.1 | TTACCGTAGGGAAAGTCGGC | ATTGGTGTCAGCAGAGGAGG |  |
| *Hdac4* | XM_052750400.1 | GTGTTTGTCAGGCTCCCTTG | TGGGTGTACTCTCCTCAGCA |  |
| *Il1β* = interleukin-1 beta, *Tnf* = tumor necrosis factor, *Icam1* = intercellular adhesion molecule 1, *Actb* = actin beta, *B2m* = beta-2-microglobulin, *Il5* = interleukin-5, *Map2k1* = mitogen-activated protein kinase kinase 1, *Plcb1* = phospholipase C beta 1, *Mknk1* = MAPK interacting serine/threonine kinase 1, *C3ar1* = complement C3a receptor 1, *Hdac4* = histone deacetylase 4. | | | |  |
|  |  |  |  |  |
|  |  |  |  |  |
|  |  |  |  |  |
